# A Rare Recurrence of Guillain-Barré Syndrome

**DOI:** 10.7759/cureus.63006

**Published:** 2024-06-23

**Authors:** Rima Othman, Mohammed Abdallah, Georges Chalouhy

**Affiliations:** 1 Critical Care and General Medicine, Faculty of Medicine, University of Balamand, Beirut, LBN; 2 Internal Medicine, University of Balamand, Beirut, LBN; 3 Intensive Care Unit, Nini Hospital, Beirut, LBN

**Keywords:** autoimmune, guillain-barré syndrome, demyelinating neurological disorder, neuropathy, lower limb weakness

## Abstract

Guillain-Barré syndrome (GBS) is an autoimmune polyneuroradiculopathy that is often characterized by progressive motor and sensory deficits. GBS often follows a particular trigger and is more common in males. Recurrent GBS is uncommon, but it can occur in 2-3% of patients with a prior GBS episode. Although recurrent GBS is generally milder than the initial episode, exceptions are possible, as illustrated in the case presented here. For both monophasic or recurrent GBS, treatment usually centers on supportive care, intravenous immunoglobulin administration, and plasmapheresis, with steroids having no impact on the prognosis. We present a case in which an 80-year-old woman with severe recurrent GBS experienced rapid deterioration within hours.

## Introduction

Guillain-Barré syndrome (GBS) is a rare acute autoimmune disease that can affect all myelinated nerves [1]. It is characterized by progressive sensory and motor deficits that reach their maximum two to four weeks after the onset of symptoms. Approximately, 75% of patients with GBS who have developed tetraplegia have symptoms of dysautonomia. Dysautonomia alone is responsible for up to 50% of the mortality associated with GBS [2]. Patients diagnosed with GBS who require mechanical ventilation have a 20% higher mortality rate compared to those without mechanical support [3]. In a large, nationwide Korean study, the disability rates in patients with GBS who were previously healthy reached up to 10% [4]. These disabilities range from a decrease in motor strength to involvement of cranial nerves; in severe cases, individuals may lose the ability to ambulate and become fully dependent on caregivers [4]. Although idiopathic cases have been reported, GBS is most commonly diagnosed after an infection [1]. The incidence is much higher in males, and the clinical presentation and the course of the disease vary greatly between patients. The diagnosis relies on electrophysiology studies and cerebrospinal fluid analysis [5]. Guidelines for treatment often include respiratory support, administration of immunoglobulins, and plasmapheresis [5]. Long-term rehabilitation is almost always required after the acute phase of GBS because the majority of the patients have lasting neurological sequelae and are left with persistent deficits [6]. Although GBS is generally monophasic, recurrences have been reported in up to 3% of previously affected patients. Risk factors for recurrence remain unknown, with the majority of recurrences having the same clinical presentation as the initial episode, but the onset is caused by a different trigger [7]. Recent studies indicate that genetic and immunologic host factors determine the patient’s risk of developing recurrent GBS, regardless of the trigger [8,9]. Meta-analysis and comparative studies have concluded that individuals with recurrent GBS are often younger than the patient in the current case and had a milder course of the primary episode [7]. These patients also tended to have a prior episode of Miller Fisher syndrome (MFS), which is a variant of GBS characterized by the triad of ophthalmoplegia, ataxia, and areflexia [7]. Recurrences of MFS are rare and are often considered to be GBS [9,10].

We present a case of recurrent severe GBS in which the patient had rapid deterioration within hours of symptom onset, without prior MFS or an identified trigger.

## Case presentation

An 80-year-old woman presented to the emergency department with upper and lower extremity weakness preceded by diarrhea two weeks earlier that required antibiotic treatment. The patient was known to have coronary artery disease, hypertension, and diabetes, and she had had an episode of GBS 17 years ago that was not preceded by infection. That episode was characterized by a gradual ascending weakness reaching the neck within weeks, but the patient did not experience respiratory compromise. She was treated promptly with intravenous immunoglobulin (IVIG), which resulted in a gradual improvement of symptoms. Physical rehabilitation was initiated, and the patient achieved near-complete recovery within three months.

**Figure 1:**
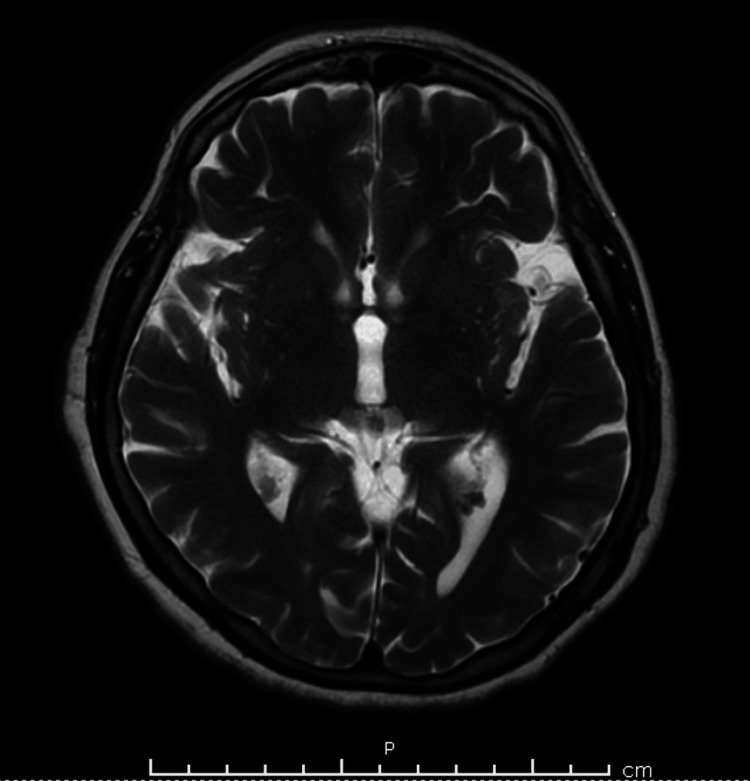
MRI of the brain showing no signs of acute bleeding and/or ischemia (plane A).

**Figure 2:**
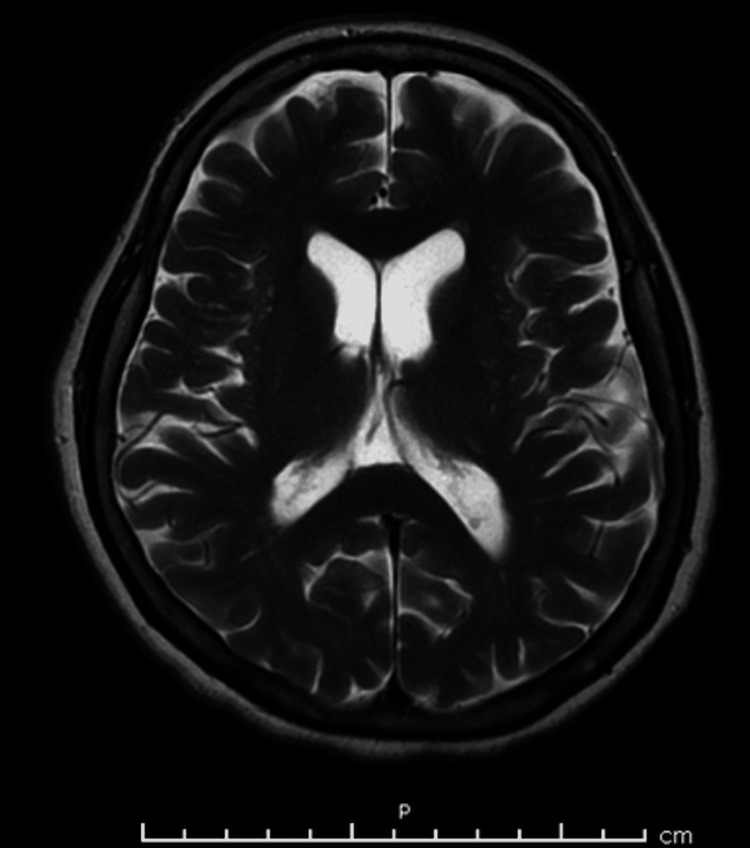
MRI of the brain showing no signs of acute bleeding and/or ischemia (plane B).

**Figure 3:**
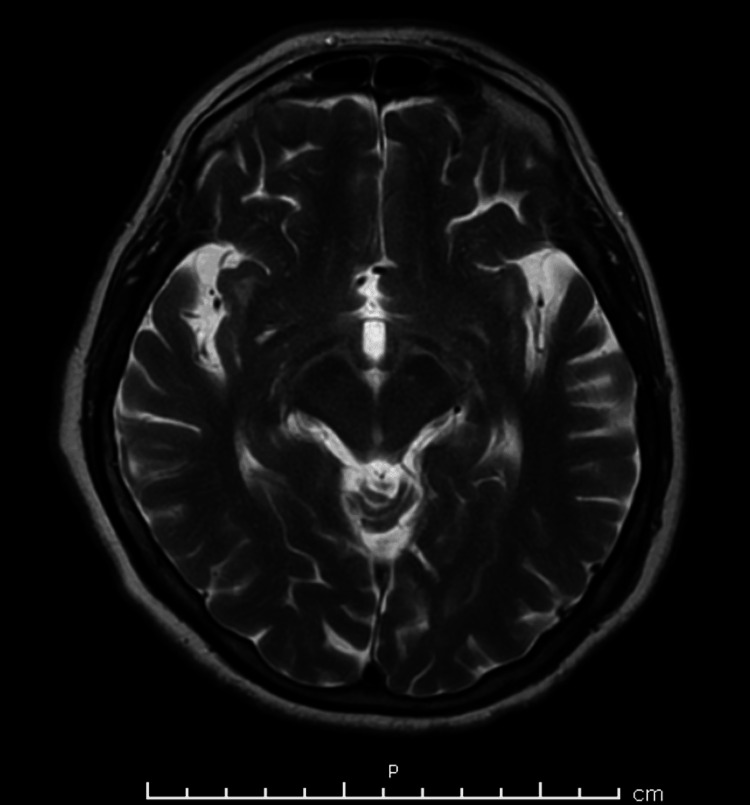
MRI of the brain showing no signs of acute bleeding and/or ischemia (plane C).

**Figure 4:**
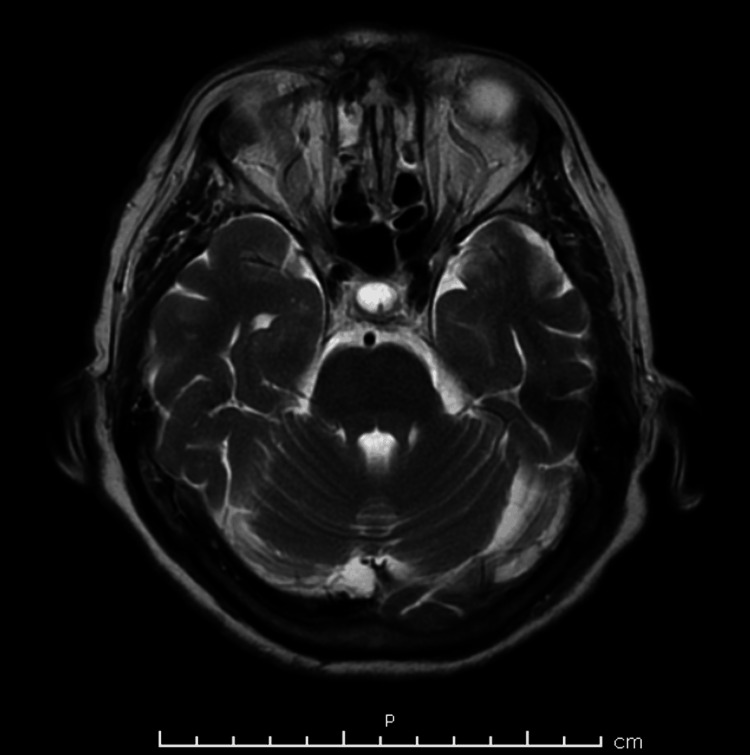
MRI of the brain showing no signs of acute bleeding and/or ischemia (plane D).

At the presentation to the emergency department, the patient had an altered mental status, and she was neither alert nor oriented. The cause of the mentioned altered mental status was not determined as no pertinent factors were deduced through history taking. Within 12 hours, the patient developed paraplegia with respiratory failure and was therefore intubated. At presentation, cerebrospinal fluid analysis revealed an elevated protein level with a normal cell count and glucose. Magnetic resonance imaging of the brain yielded normal results (Figures 1-4), and an electroencephalogram (EEG) showed normal electrical activity. Nerve conduction studies and electromyography were not done due to nonavailability.

Initial neural examination showed bilateral upper and lower motor deficits with complete areflexia, supporting the diagnosis of peripheral neuropathy GBS. No other pertinent findings in neural examination were found. The patient was diagnosed with fulminant GBS, and she was started on IVIG at a dose of 2 g/kg divided over three days. The patient’s Glasgow Coma Scale (GCS) score was 3, and she failed spontaneous breathing trials. An EEG done one week post admission showed normal electrical activity. After 15 days of intubation, the patient became a candidate for tracheostomy and percutaneous endoscopic gastrostomy (PEG) placement, both of which were done successfully. A 15-day washout period of IVIG was allowed, and as the patient’s GCS was still 3, plasmapheresis was started. The patient underwent six sessions of plasmapheresis and underwent control brain computed tomography and laboratory testing. After the six sessions were completed, the patient had a very poor prognosis and was placed on palliative care. Her hospital stay was complicated by pneumonia and a urinary tract infection that was found on admission. The patient also experienced a PEG site infection and a hypotensive episode that was nonresuscitable by fluids, requiring norepinephrine. At 110 days after admission, the patient regained her ability to spontaneously open her eyes. She then progressed during the following week to follow a finger in her visual field, blink on command, and respond to questions by blinking. During the following week (days 117-124 after admission), the patient was able to maintain spontaneous breathing through her tracheostomy and support good gas exchange to maintain normal arterial blood gas levels. Improvement was spontaneous and not related to any medication change or alternation. Physiotherapy was maintained twice daily throughout the course of her hospital stay. The patient was ultimately discharged after tracheostomy ablation. Currently, our patient has 2+ motor strength in both upper and lower extremities, reflexes are at 2+, and she can speak short phrases. The patient is continuing physiotherapy and speech therapy at a rehabilitation center.

## Discussion

With our patient's presentation, multiple differentials can be placed. We have ruled out stroke as imaging was normal. Seizures were also ruled out as EEG tracings did not show any epileptic activities, and with the history of GBS, we had little suspicion left that it was anything else other than a recurrent GBS episode. Typically, the grading of patients with GBS follows the criteria of the National Institute of Neurological and Communicative Diseases and Strokes. The GBS disability scale is a seven-point disability score, with zero points indicating no symptoms and six points indicating death [8]. Patients who are bed-bound automatically have a score of 4 or higher [8]; thus, our patient rapidly progressed to a score of 4 and above within hours of admission, and she appeared to have a very poor prognosis. Parameters for poor prognosis vary with our patient having multiple, notable of which are rapid onset of illness, severe degree of paralysis and muscle wasting, bulbar paralysis, and respiratory compromise [9]. As previously reviewed, our patient had these parameters within hours of admission and was hence in a critical state [9]. However, she spontaneously regained some muscle function after three months of intubation. Such improvement in clinical status is very rare, especially when it is independent of any medication addition or change. In addition, the literature surrounding recurrent GBS describes a subsequent episode of the disease to be milder and shorter in duration than the first [9]. The underlying pathophysiology of why the recurrence is typically milder is still unknown with many speculating that it relates to host immunological susceptibility and priming [9]. However, our patient’s GBS recurrence was notably more severe than her previous episode and progressed considerably more rapidly. Our management of the current case was difficult because there are many gaps in the understanding of GBS. The lack of knowledge on the prognosis and management of recurrent GBS is partly due to the low incidence of the condition, as well as the high mortality and disability associated with it.

## Conclusions

Although GBS is itself rare and recurs in only 2%-3% of previously diagnosed individuals, recurrent GBS can happen and may not follow the expected course, as with our patient. Recent guidelines on supportive management suggest that a poor prognosis is to be expected in such cases. However, exceptions are possible, as shown in our case. Such cases trigger many questions, and answering these questions may lead to different management plans and a much better prognosis for patients. Further research on recurrent GBS is needed to better understand the pathophysiology of the disease.
